# Therapeutic Targeting of Oxidative Stress in Hematopoietic Stem Cells: Current Strategies and Future Directions

**DOI:** 10.1155/sci/9975574

**Published:** 2026-11-10

**Authors:** Dharmaraj Senthilkumar, Sathish Kumar Venkatachalam, Nallusamy Duraisamy, Zaw Myo Hein, Vasudevan Ramachandran, Suresh Kumar, Muhammad Danial Che Ramli, Anastasiya Spaska

**Affiliations:** ^1^ Department of Life Sciences Galgotias University Greater Noida India galgotiasuniversity.edu.in; ^2^ Department of Microbiology Vinayaka Mission’s Research Foundation Salem India; ^3^ Department of Research Meenakshi Academy of Higher Education and Research Chennai India maher.ac.in; ^4^ Department of Basic Medical Sciences Ajman University Ajman UAE ajman.ac.ae; ^5^ Center of Medical and Bio-Allied Health Sciences Research Ajman University Ajman UAE ajman.ac.ae; ^6^ Department of Medical Science Faculty of Health Sciences MAIWP International University Kuala Lumpur Malaysia; ^7^ School of Pharmaceutical Sciences Vels Institute of Science Technology and Advanced Studies Chennai India vistas.ac.in; ^8^ Department of Diagnostic and Allied Health Science Management and Science University Shah Alam Selangor Malaysia msu.edu.my

**Keywords:** antioxidants, hematopoietic stem cells, oxidative stress, self-renewal, therapeutic strategies, transplantation

## Abstract

The maintenance of blood homeostasis relies heavily on hematopoietic stem cells (HSC), whose function is highly sensitive to oxidative stress caused by disrupted equilibrium between reactive oxygen species (ROS) and antioxidant systems. This review provides a comprehensive examination of the current strategies aimed at therapeutically targeting oxidative stress in HSC, outlining both the challenges and the advancements in this domain. We discuss the intricate mechanisms by which oxidative stress disrupts HSC self‐renewal, quiescence, and differentiation, as well as the implications for aging and hematological malignancies. The function of the hypoxic bone marrow microenvironment in reducing oxidative damage is highlighted alongside the metabolic adaptations that support HSC viability. Furthermore, we explore emerging therapeutic approaches, including antioxidants, gene editing technologies, and niche modulation, that aim to enhance HSC function and resilience in the face of oxidative stress. Drawing on recent preclinical and clinical findings, this review highlights the need for novel strategies that integrate a deeper understanding of redox biology with therapeutic interventions to enhance outcomes in HSC transplantation and regeneration. This integration of current findings and emerging strategies provides valuable insights for developing targeted therapies aimed at preserving HSC integrity in the treatment of hematologic diseases.

## 1. Introduction

Oxidative stress refers to a disruption in the balance between oxidant production, particularly reactive oxygen species (ROS), and the organism’s antioxidant defenses or repair mechanisms [1, 2]. This condition arises when there is an increase in ROS production or a decrease in the available antioxidant defenses, thereby disrupting the cellular redox balance [2, 3]. ROS are oxygen‐derived reactive molecules, comprising free radicals like the superoxide anion (O_2_•^−^) and hydroxyl radical (•OH), as well as nonradical species such as hydrogen peroxide (H_2_O_2_) [1–3]. These species are natural byproducts of normal cellular metabolic processes, predominantly generated in cellular compartments such as mitochondria during oxidative phosphorylation (OxPhos) and also by enzymes like NADPH oxidases, cytochrome P450, and cyclooxygenases [3–5]. While low‐to‐moderate levels of ROS are essential as signaling molecules that regulate various physiological functions, including cell proliferation, differentiation, and tissue homeostasis, their excessive accumulation can lead to detrimental effects [1, 3, 6, 7]. Elevated concentrations of ROS can cause oxidative harm to essential biomolecules like lipids, proteins, and DNA, resulting in genomic mutations, cellular senescence, and, ultimately, cell death [1, 3, 6]. Cells are equipped with sophisticated antioxidant defense systems, encompassing enzymatic antioxidants such as superoxide dismutase (SOD), catalase (CAT), and glutathione peroxidases (GPxs), as well as nonenzymatic molecules like glutathione, vitamin C, and vitamin E, which work together to neutralize excess ROS and preserve the delicate redox balance essential for cellular function [1, 5, 8].

Hematopoietic stem cells (HSC) are versatile adult stem cells positioned at the top of the blood cell development system, playing a vital role in sustaining the lifelong generation and renewal of all types of mature blood cells [4, 9–11]. These remarkable cells possess two defining properties: self‐renewal, which allows them to divide and maintain their pool, and multilineage differentiation, enabling them to give rise to all blood lineages, including erythrocytes (red blood cells), leukocytes (white blood cells), and platelets, through a process known as hematopoiesis [4, 9, 10]. The HSC typically remain in a resting state but periodically undergo division to sustain blood cell production and preserve the stem cell reservoir [4]. The study of HSC has provided foundational knowledge for understanding other stem cell systems and is critically important for regenerative medicine and clinical applications, particularly in the treatment of hematological and immune diseases [9, 12].

HSC primarily reside in specialized microenvironments within the bone marrow, often referred to as niches, which provide crucial signals that regulate their self‐renewal, quiescence, and differentiation [13–15]. A defining characteristic of these niches is their hypoxic nature, meaning that they are low in oxygen tension compared to the peripheral blood or other tissues [6, 16]. This hypoxic environment is pivotal because it inherently protects HSC from high levels of oxidative stress, which would otherwise compromise their self‐renewal capacity and functional integrity [6, 16]. HSC in these hypoxic niches predominantly rely on glycolysis for energy production. This metabolic pathway generates significantly less ROS compared to mitochondrial OxPhos, thereby contributing to the maintenance of their low ROS state [6, 16]. Maintaining a balanced redox state is essential for preserving the stem cell population and supporting immune function during both normal and stressful conditions [6]. The intricate interplay between oxidative stress and HSC function is, therefore, a fundamental aspect of hematopoietic biology, with significant implications for understanding age‐related decline, bone marrow failure syndromes, and developing therapeutic strategies for various hematological disorders and transplantation outcomes [6, 16]. Hence, in this review, we discuss and provide insight into the implications of oxidative stress on HSC, elucidate underlying molecular mechanisms, and highlight emerging therapeutic strategies aimed at mitigating oxidative damage to improve HSC function and transplantation outcomes.

### 1.1. Implications of Oxidative Stress on HSC Function

Oxidative stress significantly impacts several aspects of HSC function, including their self‐renewal, quiescence, differentiation, aging, and their involvement in various disease conditions, collectively influencing hematopoietic homeostasis [17, 18]. This section provides a comprehensive overview of these implications (Figure 1).

**Figure 1:**
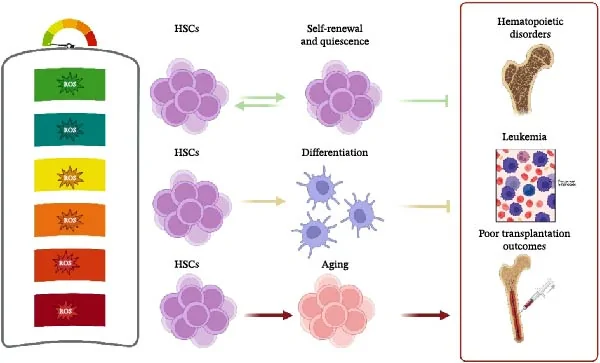
Impact of oxidative stress on HSCs function and fate. HSC, hematopoietic stem cell; ROS, reactive oxygen species. Created in BioRender. Dharmaraj, S. (2026) https://BioRender.com.

### 1.2. Self‐Renewal and Quiescence

The HSC usually remain in a quiescent state within the low‐oxygen environment of the bone marrow, where they depend mainly on glycolysis for energy, minimizing the production of ROS relative to mitochondrial OxPhos [19]. This low ROS environment is critical for protecting HSC from exhaustion and preserving their long‐term repopulating capacity and self‐renewal ability [20]. Elevated levels of ROS can stimulate HSC activity but may ultimately result in the exhaustion of the stem cell reserve [21]. For instance, Foxo3a‐deficient HSC exhibit increased ROS levels and defective quiescence maintenance, making Foxo3a^−/−^ mice sensitive to myelotoxic injury [17]. The ATM (ataxia telangiectasia mutated) protein plays a key role in maintaining HSC self‐renewal and quiescence by regulating and restricting ROS levels [17]. Conversely, supplementing with antioxidants like N‐acetyl‐L‐cysteine (NAC) can rescue the repopulating capacity of HSC and maintain quiescence by reducing ROS levels, as observed in ATM‐deficient mice [17, 22]. The ROS help regulate the delicate balance between stem cell self‐renewal and differentiation, a process essential for maintaining tissue homeostasis over the course of an organisms life [17].

### 1.3. Differentiation

The appropriate regulation of ROS levels is crucial for guiding HSC differentiation [23]. While low ROS levels are associated with HSC quiescence, increased ROS levels can drive HSC towards differentiation, particularly influencing lineage decisions in myeloid progenitors [17]. For instance, granulocyte‐monocyte progenitor cells exhibit elevated intracellular ROS levels, and in common myeloid progenitors (CMPs), higher ROS concentrations are associated with a reduced capacity to differentiate into megakaryocyte‐erythroid progenitors (MEPs) [17]. Granulocyte colony‐stimulating factor (G‐CSF), commonly administered for leukopenia treatment, stimulates HSC proliferation and differentiation, which may compromise their self‐renewal ability and risk depleting the bone marrow’s regenerative potential [17]. Increased HSC damage following G‐CSF administration is associated with elevated ROS production and activation of the p38 mitogen‐activated protein kinase (p38 MAPK) pathway [17]. Research indicates that the fate of pluripotent stem cells (PSCs) can be directly influenced by the cellular redox balance, which is, in turn, affected by metabolic changes within the PSCs [17]. High levels of intracellular ROS can lead to the loss of stem cell characteristics and encourage neuronal differentiation in models of human embryonic stem cells [24]. Therefore, understanding and modulating ROS signaling may offer novel therapeutic strategies for enhancing stem cell‐based regenerative therapies and mitigating adverse effects in clinical interventions involving hematopoietic or PSCs.

### 1.4. Aging

HSC aging is strongly associated with an increase in oxidative stress and a decline in stem cell function [25]. Aged HSC exhibit impaired self‐renewal and regenerative capacity, often linked to increased ROS levels and DNA damage. Although the number of HSC tends to increase in aged bone marrow, their functional capacity decreases significantly [26]. Aged HSC show reduced repopulation capacity in serial transplantation assays, decreased long‐term self‐renewal, heightened replicative stress, and a myeloid bias in differentiation. DNA damage responses and elevated ROS levels are considered causative factors for HSC aging [27, 28]. For instance, ATM‐deficient mice, which exhibit increased ROS levels, display functional decline in HSC and bone marrow failure, resulting in impaired self‐renewal capacity and cellular senescence [17]. Rejuvenation of aged HSC by reducing ROS levels has been demonstrated in animal models through prolonged fasting, mammalian target of the rapamycin (mTOR) inhibition with rapamycin, and upregulation of sirtuin 7 (SIRT7) [17]. Aged HSC also show higher expression of Wnt5a, and inhibiting Cdc42 activity can rejuvenate them [17]. As such, regulating ROS levels and associated signaling pathways offers a compelling avenue for combating age‐related hematopoietic dysfunction and enhancing outcomes in stem cell‐based therapies and transplantation.

### 1.5. Impacts of Oxidative Stress on Hematopoietic Disorders and Stem Cell Transplantation Outcomes

Oxidative stress is implicated in various hematopoietic disorders and significantly impacts HSC transplantation outcomes (Figure 2).

**Figure 2:**
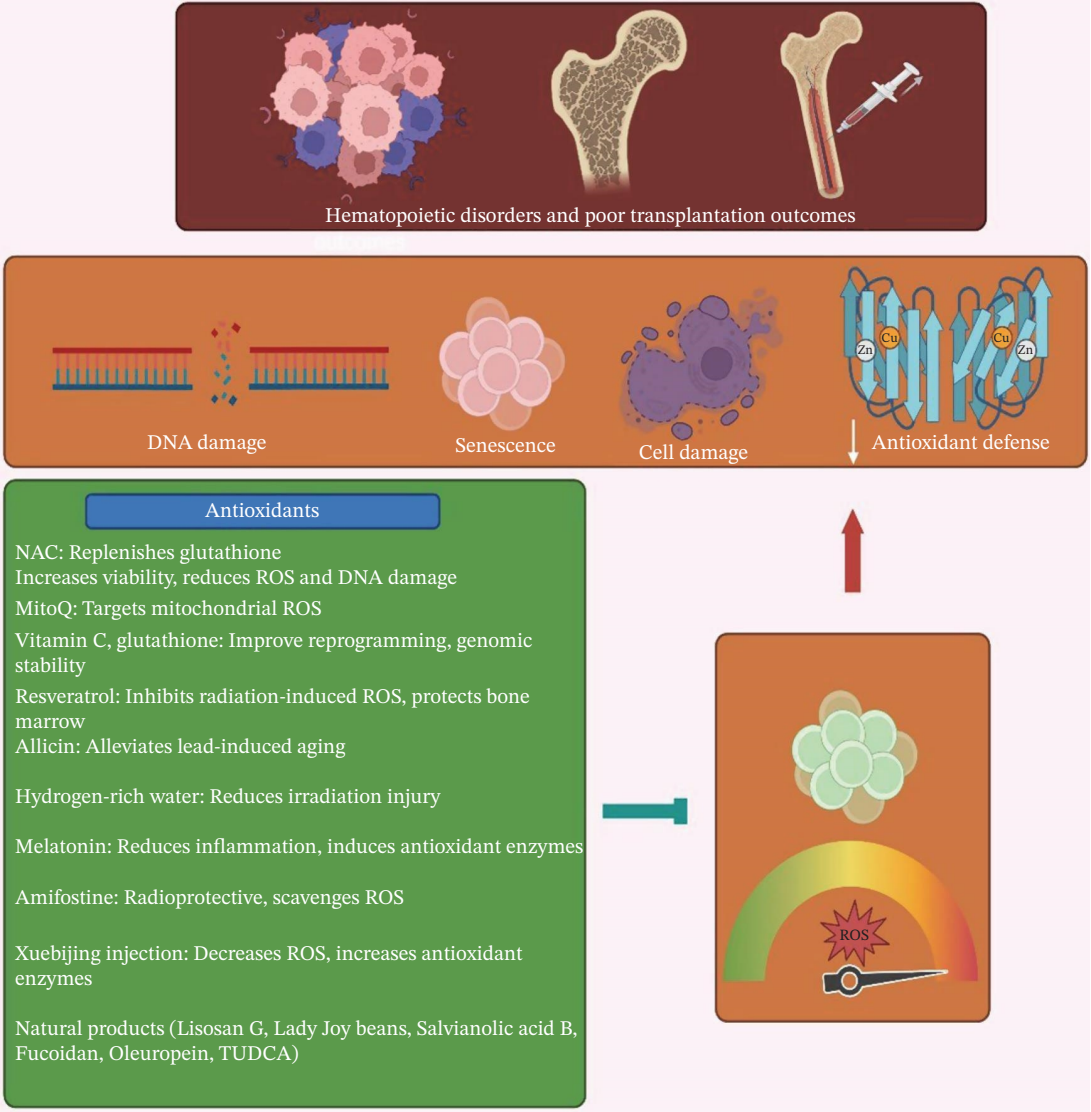
Mechanisms of oxidative stress regulation in HSCs. DNA, deoxyribonucleic acid; HSC, hematopoietic stem cell; MitoQ, mitochondria‐targeted ubiquinone; NAC, N‐acetylcysteine; ROS, reactive oxygen species; SOD, superoxide dismutase; TUDCA, tauroursodeoxycholic acid. Created in BioRender. Dharmaraj, S. (2026) https://BioRender.com.

#### 1.5.1. Bone Marrow Failure Syndromes

In inherited bone marrow failure disorders such as Fanconi anemia, hematopoietic stem and progenitor cells (HSPCs) show a sustained oxidative stress response caused by the buildup of unrepaired DNA damage [29]. Cells deficient in Fanconi anemia proteins become highly vulnerable to oxidative stress [29]. Adenylate kinase AK2, an enzyme mutated in reticular dysgenesis, safeguards the development of HSPCs against oxidative stress, and AK2 deficiency leads to increased oxidative stress in these cells [30]. Treatment with antioxidants has been shown to restore normal hematopoietic function in models lacking AK2 [30]. Bone marrow failure is associated with the failure to control elevated ROS levels and defective repair of ROS‐induced damage [31]. These approaches highlight the potential of redox modulation as a therapeutic avenue for managing inherited marrow failure syndromes and protecting HSPCs from oxidative damage‐induced exhaustion.

#### 1.5.2. Leukemia

Dysregulation of the redox environment in HSPCs can lead to the loss of signaling that typically controls cell fate, contributing to hematopoietic malignancies and the characteristics of leukemic stem cells (LSCs) [32]. Increased expression of Fbxl10 in HSCs may result in the development of myeloid or B‐lymphoid leukemia, coupled with increased cellular adenosine 5^′^‐triphosphate levels and enrichment of genes involved in mitochondrial OxPhos [33]. Emerging compounds that alter the redox balance in HSPCs and LSCs hold promise for preserving healthy HSPC function while selectively targeting malignant cells [5]. USP15, a deubiquitinating enzyme, is essential for safeguarding the genome integrity in HSC and leukemia cells, and its depletion can increase genotoxic stress [34]. Thus, modulating the redox landscape presents a promising strategy for both the prevention and treatment of hematopoietic malignancies.

#### 1.5.3. Transplantation Outcomes

Oxidative stress is a significant factor contributing to ineffective bone marrow transplantation outcomes [35]. Patients undergoing autologous hematopoietic stem‐cell transplantation (HSCT) for multiple myeloma and lymphoma exhibit a hyperoxidative state with a high DNA damage index (DI) compared to healthy controls, and the conditioning regimen exacerbates this condition [36]. This hyperoxidative state is also reflected in significantly high basal levels of the lipid peroxidation product malondialdehyde (MDA) and altered antioxidant enzyme activities, such as SOD [36]. Higher basal levels of nitric oxide metabolites (NOx) and the DNA DI are positively correlated with longer grafting times in lymphoma patients. In comparison, lower levels of GPx are associated with longer grafting times in multiple myeloma patients, indicating that these biomarkers can predict toxicity and grafting time [36]. The HSC harvested under nonphysiological ambient air conditions lose their ability for long‐term repopulation, a consequence of ROS generation associated with cyclophilin D (CypD) and the mitochondrial permeability transition pore (MPTP) [17]. Inhibition of CypD can reduce ROS and enhance the yield and transplantation efficacy of HSC [17]. Enhanced blood vessel permeability may result in elevated ROS levels and promote greater migration and differentiation of HSPCs by allowing plasma to carry ROS‐inducing factors [17]. Hyperbaric oxygen therapy (HBOT) is being investigated for its role in improving hematopoietic stem/progenitor cell homing, engraftment, and immune reconstitution [37]. Together, these approaches highlight the importance of redox control in optimizing HSCT outcomes and minimizing transplantation‐associated complications.

### 1.6. Mechanisms of Oxidative Stress in HSCs

The functional integrity and fate decisions of HSC are intimately regulated by the cellular oxidation‐reduction state, where an imbalance favoring ROS over antioxidant defenses leads to oxidative stress [38]. ROS, including superoxide, hydrogen peroxide, and hydroxyl radicals, are continuously generated as natural by‐products of normal cellular metabolism, primarily through aerobic processes within mitochondria [39, 40]. However, the tight regulation of ROS levels is critical as excessive accumulation beyond the capacity of cellular antioxidant systems can lead to detrimental effects, including stem cell death, aging, genomic mutations, proliferation, and differentiation [39]. Conversely, low‐to‐moderate amounts of ROS act as vital signaling molecules that control key physiological and cellular functions, including the regulation of stem cell self‐renewal and differentiation. Gaining insight into these processes is important for advancing regenerative medicine and treating a range of blood‐related diseases [39]. Understanding the molecular mechanisms that govern redox homeostasis in HSC not only deepens our knowledge of stem cell physiology but also opens promising avenues for therapeutic interventions aimed at preserving HSC integrity, enhancing transplantation outcomes, and targeting LSCs in blood cancers (Table 1).

**Table 1:** Enhancing antioxidant defenses in HSCs: mechanisms, strategies, and implications.

Strategy category	Intervention/compound	Primary mechanism/target	Outcome on HSCs	Implications	References
Pharmacological activation	DDO1002 (NRF2‐KEAP1 inhibitor)	Activates NRF2‐ARE pathway, upregulates antioxidant genes (HO‐1, GST)	↓ ROS, ↓ senescence, ↑ self‐renewal, ↑ reconstitution capacity	Promising therapy for age‐related hematologic decline	[41]
	Sulforaphane	Indirect NRF2 activator	↑ Antioxidant enzymes (NQO1, HO‐1), ↑ engraftment in transplants	Nutraceutical approach for oxidative stress modulation	[5, 18]
	Cyclosporine A	Inhibits MPTP, preserves mitochondrial function	Protects against oxidative shock, ↑ posttransplant recovery	Supports procedural optimization during cell handling	[42]
	Tempol	SOD mimetic, scavenges superoxide radicals	Maintains HSC quiescence and clonogenicity during culture	Useful in ex vivo HSC expansion	[5]
Dietary/natural compounds	*Angelica sinensis* polysaccharides (ASP)	Antioxidant + niche modulator ↓ SASP, ↑ gap junction communication	↓ DNA damage, ↑ HSC function in aging models	Herbal medicine with regenerative potential	[43]
	Ascorbic acid 2‐phosphate (AA2P)	Stable vitamin C derivative, direct ROS scavenging	↑ Proliferation, ↑ migration, ↑ engraftment	Enhances transplantation outcomes	[16, 36]
	N‐acetylcysteine (NAC)	Glutathione precursor, inhibits TNF‐α/ROS axis	Preserves HSC quiescence, ↑ survival under stress	Widely studied antioxidant with translational relevance	[18, 30, 44]
	Resveratrol, Curcumin	Anti‐inflammatory and antioxidant	↓ ROS and inflammaging	Adjunctive therapy to support healthy aging of HSCs	[45]
Niche restoration	ASP + BMSC modulation	Restores niche homeostasis, enhances osteogenic balance	Improves HSC support environment	Combats age‐related niche inflammation and adipogenesis	[43]
Gene and molecular engineering	SOD/catalase overexpression	Enhanced antioxidant enzyme expression	↑ HSC survival and engraftment	Gene therapy approach for oxidative resilience	[22, 46]
	HIF‐based preconditioning	Mimics hypoxia → boosts endogenous antioxidant responses	↑ Antioxidant capacity, ↑ regenerative function	Enhances transplant efficiency and HSC quality	—
Environmental optimization	Hypoxic collection/processing	Reduces oxygen exposure and ↓ ROS production	↑ HSC survival, ↑ engraftment	Standardization in clinical HSC processing protocols	[16]
Disease‐specific modulation	NAC in transplantation	Replenishes glutathione; limits ROS accumulation during conditioning	Protects against apoptosis, ↑ reconstitution potential	Applicable in inflammatory hematologic disorders (e.g., MDS, anemia)	[30, 36, 47]
	Pro‐oxidant targeting of LSCs	↑ ROS selectively in leukemic cells	Induces apoptosis of LSCs	Emerging dual‐therapy model: antioxidants for HSCs + ROS inducers for LSCs	[30, 44]
Pathophysiology insights	NRF2‐KEAP1 pathway (declines with age)	Loss leads to increased vulnerability to ROS	↑ Susceptibility to DNA damage, ↓ self‐renewal	Key biomarker and target for aging‐related interventions	[15, 48]

*Note:* Arrows denote an increase (up) or a decrease (down) relative to untreated control.

Abbreviations: AA2P, ascorbic acid 2‐phosphate; ARE, antioxidant response element; ASP, *Angelica sinensis* polysaccharides; BMSC, bone marrow stromal cell; GST, glutathione S‐transferase; HIF, hypoxia‐inducible factor; HO‐1, heme oxygenase‐1; HSC, hematopoietic stem cell; KEAP1, Kelch‐like ECH‐associated protein 1; LSC, leukemic stem cell; MDS, myelodysplastic syndrome; MPTP, mitochondrial permeability transition pore; NAC, N‐acetylcysteine; NQO1, NAD(P)H quinone dehydrogenase 1; NRF2, nuclear factor erythroid 2‐related factor 2; ROS, reactive oxygen species; SASP, senescence‐associated secretory phenotype; SOD, superoxide dismutase; TNF‐alpha, tumor necrosis factor alpha.

### 1.7. Metabolic Regulation and ROS Production

HSCs sustain low ROS levels by inhabiting the low‐oxygen environment of the bone marrow, a condition essential for maintaining their self‐renewal ability and shielding them from oxidative damage [5, 39]. This hypoxic niche ensures that HSC predominantly rely on glycolysis for energy production. This anaerobic metabolic pathway is significantly less efficient for ATP generation compared to OxPhos but is highly effective at maintaining low intracellular ROS levels [5, 22, 39]. In contrast, typically, quiescent differentiated cells utilize OxPhos, which generates higher levels of ROS [39].

During the process of differentiation, HSC undergo a metabolic shift, transitioning from a glycolysis‐dependent state to one that increasingly relies on mitochondrial oxidative metabolism (OxPhos) [22, 39]. This shift supports the increased energy demands of differentiating cells, but it also concomitantly increases the production of ROS [22, 39]. For example, the differentiation of PSCs into functional cardiomyocytes requires a conversion to preferentially use mitochondrial‐mediated oxidative metabolism preferentially, highlighting the critical role of mitochondrial‐dependent energetic circuits in cell fate decisions [39]. This metabolic shift and the resulting increase in ROS are not merely by‐products but function as upstream signals that actively promote HSC differentiation and lineage commitment [22]. Granulocyte‐monocyte progenitor cells exhibit elevated intracellular ROS levels, which inversely correlate with the ability of CMPs to differentiate into MEPs.

Beyond mitochondrial respiration, other intracellular sources contribute to ROS production in the HSC. NADPH oxidase 4 (Nox4) is involved in generating H_2_O_2_ signaling that drives PSC differentiation toward a vascular smooth muscle cell phenotype [39]. Additionally, enzymes such as peroxisomal enzymes, microsomal cytochrome P450 enzymes, and flavoprotein oxidases can generate ROS as by‐products of their catalytic activities [40]. The fine control of these metabolic shifts and the associated ROS levels is therefore central to maintaining HSC function, influencing their self‐renewal, and guiding their precise differentiation into mature blood lineages [39].

### 1.8. Role of Transcription Factors

Transcription factors are critical regulators that modulate the oxidative stress response in HSC, influencing their fate and maintaining genomic stability. The Forkhead box O (FOXO) family of transcription factors, particularly FOXO3, is essential for maintaining HSC quiescence, self‐renewal, and protecting them from oxidative damage [29, 39, 49, 50]. FOXO3 achieves this by regulating the expression of genes involved in repairing oxidative DNA damage, thereby safeguarding HSC genomic stability [29]. For instance, loss of FOXO3 in mouse models leads to significantly elevated ROS levels and the accumulation of oxidative DNA damage in HSPCs under homeostatic conditions [29, 49]. These FOXO3‐deficient HSPCs also exhibit compromised DNA repair mechanisms, particularly impacting the base excision repair (BER) pathway, which plays a vital role in fixing oxidative DNA damage. Treatment with antioxidants, such as NAC, can partially rescue some of these defects, including normalizing cell cycle parameters and reducing DNA damage in FOXO3‐deficient cells [29].

Another pivotal transcription factor is nuclear factor (erythroid‐derived 2)‐related factor 2 (Nrf2), functioning as the key regulator of the cell’s antioxidant defense system [51]. During oxidative stress, Nrf2 separates from its inhibitor, Kelch‐like ECH‐associated protein 1 (Keap1), and moves into the nucleus [51]. Within the nucleus, it attaches to antioxidant response elements (AREs) located in the promoter regions of specific genes, triggering the expression of various antioxidant and detoxifying enzymes, thus restoring redox homeostasis and protecting the HSC from damage [51]. Nrf2 activity is also involved in regulating the expression of CXCR4, a receptor crucial for HSC retention in the bone marrow niche, demonstrating a link between antioxidant responses and HSC localization.

Hypoxia‐inducible factors (HIFs), particularly HIF‐1α, play a key role in regulating HSC metabolism and ROS levels. HIF‐1α is activated in HSC, facilitating a shift in cellular metabolism from mitochondrial respiration to glycolysis, a process that limits ROS production. This metabolic adaptation mediated by HIF‐1α is essential for maintaining HSC function and their long‐term repopulation capacity. Without functional HIF‐1α, HSCs lose their ability to regulate ROS, and their long‐term repopulation capacity is compromised. Collectively, these transcription factors form an intricate regulatory network that senses and responds to redox challenges, ensuring the proper maintenance and function of the HSC.

### 1.9. Signaling Pathways

Oxidative stress in HSC activates several crucial signaling pathways that mediate cellular responses, influencing HSC fate decisions, proliferation, differentiation, and aging [22]. The p38 MAPK pathway is a prominent responder to increased ROS levels in HSC. Activation of p38 MAPK limits the lifespan of HSC in vivo and promotes premature aging and senescence [44]. This occurs partly by upregulating cyclin‐dependent kinase (CDK) inhibitors, such as p16Ink4a and p19Arf, which induce cellular senescence [52]. Studies have demonstrated that inhibiting p38 MAPK with agents such as SB203580 can rescue ROS‐induced defects in HSC repopulating capacity and help maintain HSC quiescence [5, 53].

The mTOR pathway is also significantly modulated by oxidative stress in HSC. Increased mTOR activity has been observed in aged HSC and is linked to reductions in hematopoiesis and lymphopoiesis. High ROS levels can directly affect mTOR signaling, contributing to functional decline in HSC [52]. Conversely, pharmacological inhibition of mTOR activity using rapamycin has been shown to enhance the lifespan of aged mice and improve the repopulation capacity of aged HSCs, suggesting its role in mitigating ROS‐induced dysfunction and rejuvenating HSC [46]. These therapeutic strategies highlight the potential of targeting redox‐regulated signaling networks to protect HSC from oxidative stress, offering avenues for enhancing stem cell maintenance and improving outcomes in aging, transplantation, and hematological disease contexts.

## 2. Therapeutic Strategies Against Oxidative Stress in HSCs

The functional integrity and fate decisions of HSC are intimately regulated by the cellular oxidation‐reduction (redox) state, where an imbalance favoring ROS over antioxidant defenses leads to oxidative stress [36]. Although low to moderate ROS levels act as important signaling molecules that govern physiological and biological processes, they play a key role in regulating stem cell self‐renewal and differentiation, excessive accumulation beyond the capacity of cellular antioxidant systems can lead to detrimental effects such as affecting aging, stem cell death, genomic mutations, proliferation, and differentiation [18, 36]. Therefore, developing therapeutic strategies to mitigate oxidative stress in HSC is crucial for preserving HSC function, enhancing transplantation outcomes, and treating hematopoietic disorders [16, 30]. These strategies encompass pharmacological agents, environmental and procedural controls, and gene therapy approaches (Table 1).

### 2.1. Antioxidant Supplementation

Direct supplementation with antioxidants is a primary strategy employed to scavenge excess (ROS) and protect HSC from oxidative damage [16, 18]. This approach aims to restore redox homeostasis by neutralizing free radicals and augmenting the cellular antioxidant defense system [18].

NAC is a well‐researched antioxidant known for its ability to restore intracellular glutathione, an essential part of the cells antioxidant defense mechanism [18, 30]. Preclinical studies have demonstrated that NAC supplementation can significantly increase HSC viability, lower intracellular ROS levels, and diminish DNA damage in murine HSPCs cultured outside the body, without impairing their ability to commit to specific cell lineages [30, 44]. NAC treatment has also been shown to rescue the repopulating capacity of ATM‐deficient HSC, thereby preventing bone marrow failure [54]. Moreover, in induced PSC (iPSC) reprogramming, NAC supplementation enhances efficiency and reduces genomic aberrations by preventing oxidative damage [18, 36]. It can also increase cellular proliferation and genomic stability in bone marrow mesenchymal stem cells (BMSCs) cultured at normoxic conditions [18].

Mitochondria‐targeted ubiquinone (MitoQ) is a specialized antioxidant engineered to accumulate within mitochondria, which are major intracellular sources of ROS [18]. MitoQ efficiently rescues and improves PSC stemness by modulating mitochondrial ROS levels, particularly by preventing superoxide production within the mitochondria [18].

Other antioxidants are also important contributors to reducing oxidative stress. Vitamin C (ascorbic acid) and glutathione enhance reprogramming efficiency and maintain genomic stability by neutralizing ROS [16, 36]. Resveratrol, a potent antioxidant, can significantly inhibit radiation‐induced increases in ROS production in HSC and ameliorate long‐term bone marrow injury [45]. Allicin, extracted from garlic, has been shown to alleviate lead‐induced HSC aging phenotypes [18]. Hydrogen‐rich water can ameliorate total body irradiation‐induced HSC injury by reducing hydroxyl radicals and mitigating DNA damage [42]. Melatonin exhibits protective effects against inflammation in neural stem cells, reducing nitric oxide production and stimulating the expression of antioxidant enzymes [18]. Amifostine, an FDA‐approved radioprotective drug, acts as an ROS scavenger to protect primitive hematopoietic progenitors against chemotherapy cytotoxicity [16]. Furthermore, natural products such as Lisosan G (LG) and Lady Joy (LJ) beans have been shown to increase endothelial progenitor cells (EPCs) viability, protect against oxidative stress, enhance adhesion, reduce senescence, and decrease ROS generation [16]. Salvianolic acid B (SalB) protects bone marrow EPCs from oxidative stress, increasing their migration ability and tube formation capacity [16]. Fucoidan, a sulfated polysaccharide, possesses antioxidant properties and enhances EPC function, aiding in the repair of damaged blood vessels [16]. Tauroursodeoxycholic acid (TUDCA) has been shown to reduce ROS levels, increase progenitor cells, and promote the differentiation of CD34+ HSC into EPCs [16]. It is crucial to note that while beneficial, the appropriate dose of antioxidant supplementation is critical, as excessive levels can paradoxically suppress ROS to subphysiological levels, potentially downregulating DNA repair enzymes and leading to genomic instability [18]. Antioxidant supplementation, as part of a broader redox‐modulating strategy, holds significant promise for improving outcomes in bone marrow transplantation, mitigating age‐related hematopoietic decline, and treating inherited and acquired hematologic disorders (Table 1).

### 2.2. Pharmacological Modulation of Signaling Pathways

Targeting key redox‐sensitive signaling pathways offers a precise approach to mitigate oxidative stress and its downstream detrimental effects on HSC, thereby preserving their function and viability [55]. The p38 MAPK pathway is significantly activated by increased ROS levels in HSC, limiting their lifespan and promoting senescence [30]. Inhibition of p38 MAPK with agents like SB203580 has been shown to rescue ROS‐induced defects in HSC repopulating capacity and maintain HSC quiescence [56]. This approach counteracts the negative effects of p38 MAPK activation by high ROS levels, which otherwise accelerate HSC aging [36].

Sirtuins (SIRT), particularly SIRT1 and SIRT3, are NAD‐dependent deacetylases that regulate stem cell function, aging, and oxidative stress responses [22, 48]. Upregulating SIRT3 has been shown to protect aged human mesenchymal stem cells (hMSCs) against oxidative stress, enhance their efficacy in cell therapy for ischemic heart diseases, and improve the regenerative capacity of aged HSC by regulating mitochondrial metabolic homeostasis and reducing ROS [22]. SIRT1 activation, for instance, by resveratrol, has also been shown to ameliorate radiation‐induced chronic oxidative stress in HSC [45]. SIRT1 inhibition is also being explored as a novel approach for the ablation of LSCs [48]. Other signaling modulators include the inhibition of Cdc42 activity, which, when inhibited by agents like CASIN, has been reported to rejuvenate aged HSC that typically show higher expression of Wnt5a [57]. The transcription factor FOXO3 is essential for maintaining HSC pluripotency and protecting them from oxidative stress, partly by regulating genes involved in the repair of oxidative DNA damage [57]. These findings underscore the potential of redox‐targeted therapies not only in delaying HSC aging and improving transplantation outcomes but also in selectively eliminating LSCs. Continued exploration of these signaling networks will be key to developing precise, dual‐purpose treatments for hematopoietic dysfunction and malignancy.

### 2.3. Environmental and Procedural Control

Optimizing the microenvironment and handling procedures for HSC is crucial for significantly reducing oxidative stress during their collection, culture, and transplantation [30]. These interventions aim to mimic the native low‐oxygen conditions of the bone marrow niche and to minimize ROS generation during ex vivo manipulation. Hypoxic conditions are fundamental for preserving HSC function [30, 36]. HSCs physiologically reside in a hypoxic microenvironment (1%–4% oxygen) within the bone marrow, which naturally maintains low intracellular ROS levels and supports HSC expansion and self‐renewal [30, 36, 54]. When HSC are exposed to normoxic conditions (21% oxygen) during ex vivo expansion or maintenance, they experience increased oxygen tension and enhanced ROS production, which may disrupt their self‐renewal abilities and encourage differentiation, resulting in early senescence and a reduction in functional stem cell numbers [30]. Therefore, culturing and handling HSC under hypoxic conditions (e.g., 1%–5% O_2_) rather than ambient air conditions significantly boosts the efficiency of iPSC generation and enhances both the quantity and functionality of HSC [36]. This controlled hypoxic environment helps maintain low ROS levels, preserving HSC self‐renewal and protecting them from oxidative damage [36]. Antioxidant supplementation in culture media is a key procedural intervention. The addition of antioxidants like NAC to culture media actively scavenges ROS, improves cell viability, reduces DNA damage, and sustains lineage commitment potential without compromising differentiation capacity [30]. NAC replenishes intracellular glutathione, counteracting the oxidative stress induced by normoxic culture conditions [30]. Pharmacologic inhibition of mitochondrial pathways involved in ROS production is also employed. For example, inhibition of CypD using cyclosporine A (CsA) can antagonize MPTP induction, thereby reducing ROS and enhancing the yield and transplantation efficacy of HSC [42]. This strategy is particularly beneficial during HSC collection from bone marrow or cord blood, processes that involve exposure to air and increased ROS production [42]. Furthermore, tight control of culture environment parameters, including temperature and media composition enriched with appropriate growth factors and nutrients, aids in modulating endogenous ROS generation and supporting HSC maintenance [30]. Maintaining these optimized conditions during HSC expansion or processing is vital to mitigate oxidative stress and ensure their therapeutic potential (Figure 3) [30]. Together, these approaches underscore the importance of recreating physiological oxygen levels, supplementing with antioxidants, and modulating mitochondrial ROS production to preserve the functional integrity of HSC during clinical and research applications.

**Figure 3:**
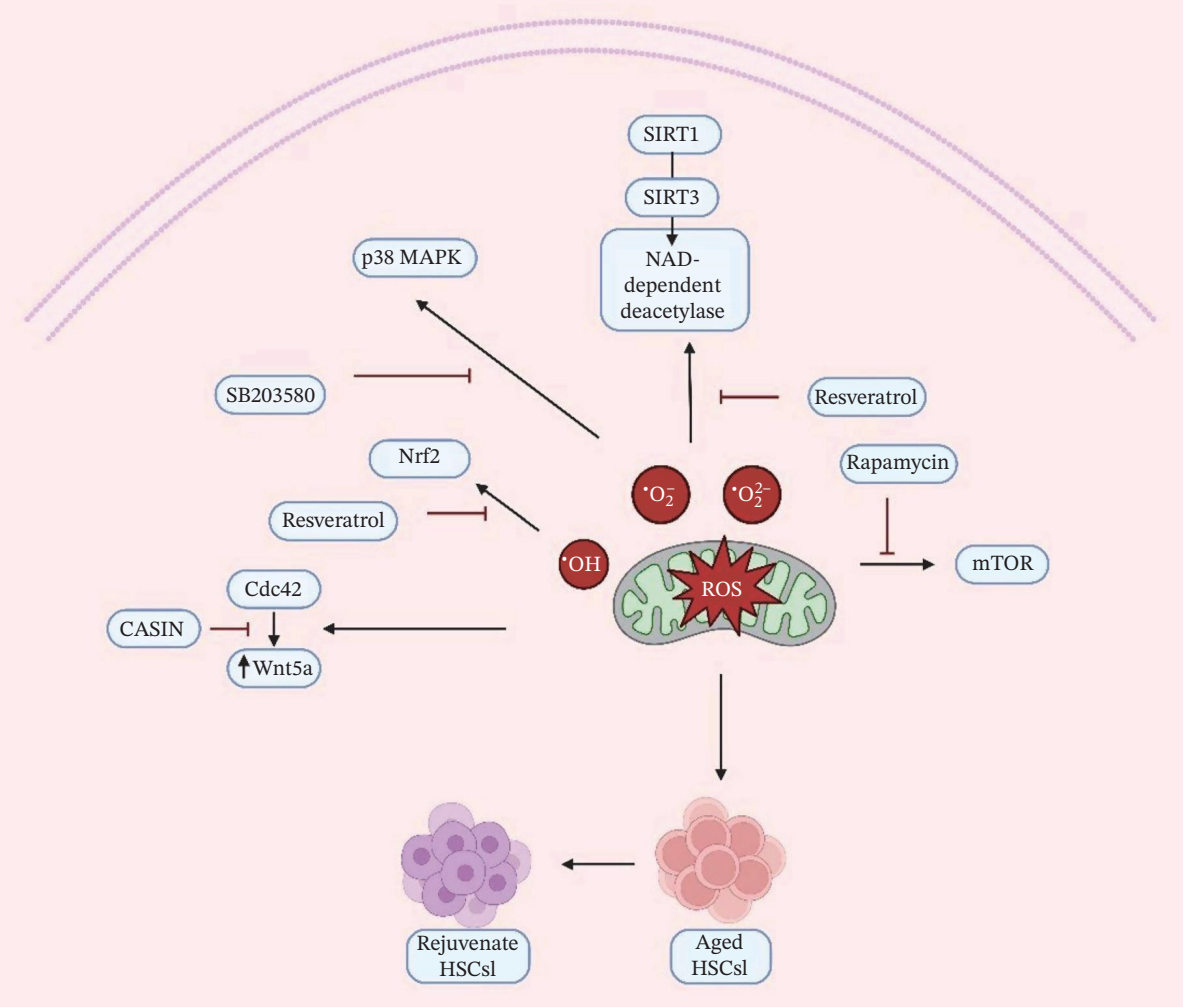
Schematic representation of the protective mechanisms of antioxidant supplementation on HSCs. Antioxidants scavenge excess ROS and restore redox balance, thereby enhancing cellular antioxidant defenses and mitigating oxidative damage to HSCs. CASIN, Cdc42 activity‐specific inhibitor; Cdc42, cell division cycle 42; HSC, hematopoietic stem cell; mTOR, mammalian target of rapamycin; NAD, nicotinamide adenine dinucleotide; NRF2, nuclear factor erythroid 2‐related factor 2; p38 MAPK, p38 mitogen‐activated protein kinase; ROS, reactive oxygen species; SIRT, sirtuin; Wnt5a, Wnt family member 5A. Created in BioRender. Dharmaraj, S. (2026) https://BioRender.com.

### 2.4. Gene Therapy Approaches

Gene therapy represents an innovative frontier for enhancing the intrinsic antioxidant capacity of HSC and correcting genetic predispositions that contribute to oxidative stress‐related defects [46]. This approach aims to provide a durable solution by modifying the cells themselves to withstand oxidative challenges better (Figure 4). One promising strategy involves the overexpression of antioxidant‐regulating genes within the HSC. For instance, Sirtuin3 (SIRT3) plays a critical role in mitochondrial metabolic homeostasis and is associated with reduced oxidative damage [22]. Studies have shown that the overexpression of SIRT3 in aged hMSCs increases the levels of antioxidant enzymes, such as manganese SOD (MnSOD) and CAT, and improves cell survival under oxidative stress, suggesting similar benefits for HSC [22]. Another approach focuses on correcting mitochondrial dysfunction through gene therapy as mitochondria are significant sources of ROS [58]. In models of mitochondrial encephalopathies, lentiviral vectors have been developed to overexpress specific mitochondrial genes (e.g., Coq9 mRNA and COQ9 protein) in hematopoietic progenitor cells, which successfully restored mitochondrial function and cellular fitness [58]. Such strategies could be adapted to address mitochondrial defects in HSC that render them more susceptible to oxidative stress.

**Figure 4:**
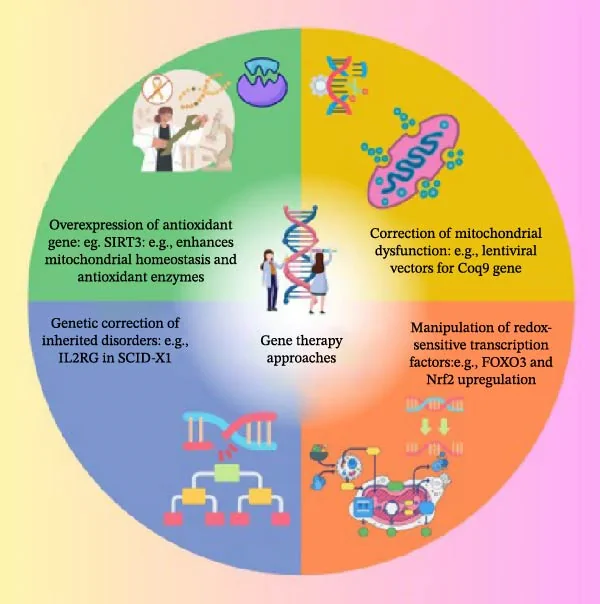
Gene therapy strategies to enhance the intrinsic antioxidant capacity of HSCs and address genetic predispositions associated with oxidative stress‐related defects. Coq9, coenzyme Q9 homolog; FOXO3, forkhead box O3; HSC, hematopoietic stem cell; NRF2, nuclear factor erythroid 2‐related factor 2; ROS, reactive oxygen species. Created in BioRender. Dharmaraj, S. (2026) https://BioRender.com.

Manipulation of redox‐sensitive transcription factors through genetic means also holds potential [55]. For instance, the FOXO family of transcription factors (e.g., FOXO3) is crucial for protecting HSC from oxidative damage and maintaining genomic stability [57]. Upregulating the activity or expression of these factors via gene therapy could enhance the cells natural antioxidant defenses and DNA repair mechanisms [57]. Similarly, targeting Nrf2, a master regulator of antioxidant enzymes, could boost the expression of a wide array of protective proteins, providing a robust defense against oxidative damage in HSC [45]. Furthermore, genetic correction of inherited disorders where HSC are inherently more sensitive to oxidative stress, such as Fanconi anemia, could profoundly improve their resilience [46]. By correcting the underlying genetic mutations, gene therapy aims to restore the cells’ ability to manage oxidative stress and DNA damage, thereby improving hematopoietic function [46]. Lentiviral vectors are often utilized for efficient and targeted gene delivery into HSC‐enriched populations, aiming to enhance transduction rates while preserving essential stem cell properties, which is vital for successful therapeutic outcomes [58]. While still primarily in preclinical stages, these gene therapy‐based interventions offer a powerful and potentially long‐lasting solution for protecting HSC from oxidative stress and enhancing their therapeutic efficacy.

### 2.5. Clinical and Preclinical Evidence

Extensive clinical and preclinical research has evaluated the efficacy of various therapeutic strategies aimed at mitigating oxidative stress in HSC, with promising results across different approaches [16, 54]. These studies collectively underscore the importance of oxidative stress management for preserving HSC viability and function and enhancing transplantation outcomes.

Antioxidant supplementation has been a major focus of research. Preclinical studies consistently show that NAC significantly improves HSC function. For instance, NAC supplementation increased cell viability, decreased intracellular rROS levels, and reduced DNA damage in murine HSPCs cultured ex vivo without negatively impacting their lineage commitment potential [30]. NAC also restored the repopulating capacity of ATM‐deficient HSC, preventing bone marrow failure in preclinical models [54]. Beyond NAC, studies have demonstrated that LG and LJ beans increase EPCs viability and shield them from damage caused by oxidative stress, improving adhesion and reducing senescence [16]. SalB enhanced the migration and tube formation capacity of bone marrow EPCs under oxidative stress [16]. Fucoidan restored the expression of key functional surface markers and stimulated tube formation in senescent EPCs, leading to improved blood vessel repair in a murine ischemia model after transplantation of preconditioned EPCs [16]. TUDCA increased progenitor cell populations and promoted differentiation of CD34+ HSC into EPCs, ultimately leading to blood vessel regeneration in an ischemic hindlimb model [16].

Pharmacological modulation of redox‐sensitive signaling pathways has also shown efficacy. Inhibition of p38 MAPK, a pathway activated by increased ROS, has been demonstrated to rescue ROS‐induced defects in HSC repopulating capacity and maintain quiescence in preclinical settings [56]. Similarly, pharmacological inhibition of the mTOR pathway by rapamycin has been shown to enhance the lifespan of aged mice and improve the repopulation capacity of aged HSC [57]. These interventions highlight the potential to rejuvenate HSC and enhance their regenerative potential.

Environmental and procedural control during HSC handling has significant preclinical evidence supporting its benefits (Figure 5). Maintaining hypoxic conditions during HSC collection and culture has been shown to minimize oxidative stress by sustaining low ROS levels, preserving HSC quiescence, and enhancing the yield and long‐term repopulating potential of HSC compared to normoxic conditions [30, 36]. Furthermore, pharmacologic inhibition of CypD by cyclosporine A reduced ROS production during ex vivo handling, which has been linked to enhanced HSC yield and transplantation efficacy [42].

**Figure 5:**
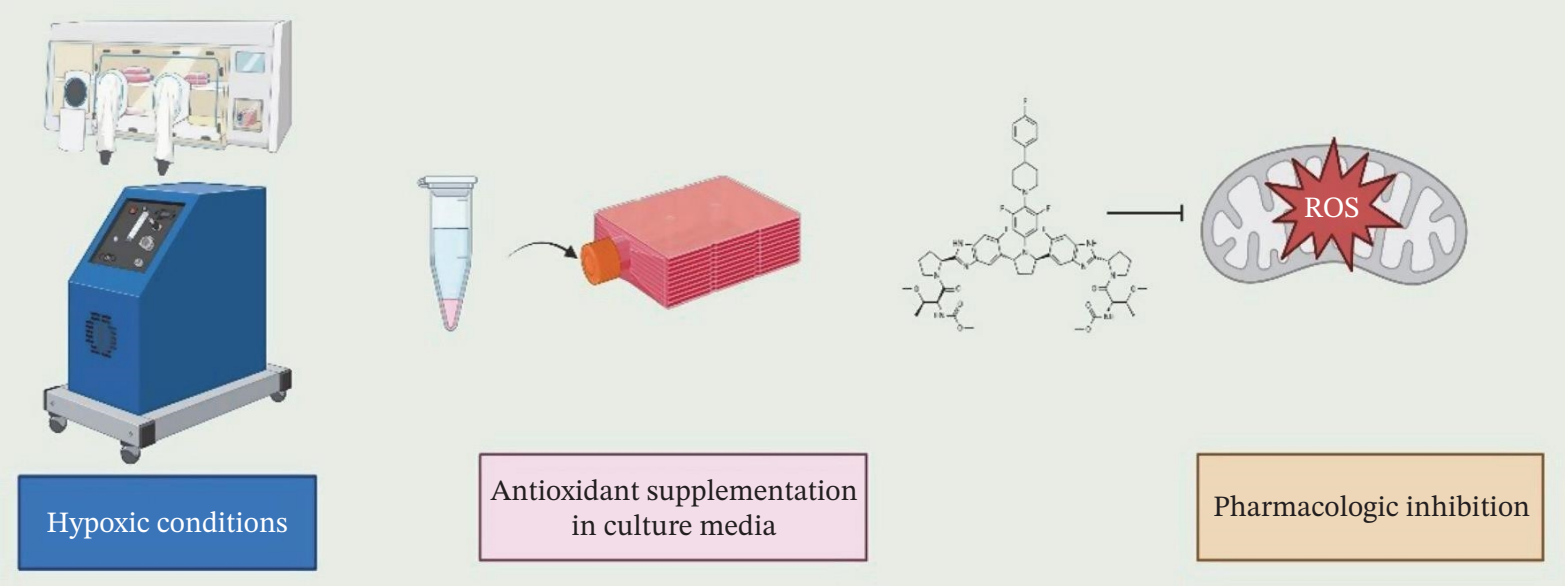
Optimizing the microenvironment and handling procedures for HSCs is essential to significantly minimize oxidative stress during their collection, culture, and transplantation. HSC, hematopoietic stem cell; ROS, reactive oxygen species. Created in BioRender. Dharmaraj, S. (2026) https://BioRender.com.

While gene therapy approaches are largely in the preclinical development stage, studies have shown promise. In preclinical models, elevated expression of SIRT3 safeguarded aged hMSCs from oxidative stress and enhanced their therapeutic effectiveness in treating ischemic heart disease [22]. Lentiviral vectors designed to correct mitochondrial dysfunction have also shown success in restoring mitochondrial function and cellular fitness in hematopoietic progenitor cells from relevant animal models [58]. This evidence points to a future role for genetic interventions in bolstering HSC antioxidant defenses. Taken together, these studies strongly support the conclusion that tailored antioxidant therapy, pharmacological targeting of oxidative stress signaling, and optimized environmental conditions effectively mitigate ROS‐induced damage in HSC, thereby improving their therapeutic potential and transplantation outcomes in various disease contexts. Continued research in these areas is crucial for translating these promising findings into clinical practice [16, 18, 30].

A mechanistically distinct strategy targets the redox‐active iron pool itself rather than the ROS it generates. Myeloablative conditioning transiently arrests erythropoiesis and releases a burst of iron into plasma, saturating transferrin and generating nontransferrin‐bound iron (NTBI), a species capable of catalyzing hydroxyl radical formation through Fenton chemistry. In hematological stem cell transplant recipients, a single intravenous dose of human apotransferrin bound this free iron effectively and restored the iron‐binding capacity of plasma [59]. Repeated apotransferrin administration during myeloablative conditioning and allogeneic transplantation maintained iron in the transferrin‐bound form in a proportion of recipients, although full suppression of NTBI was not achieved at the doses tested, indicating that dose optimization remains an unresolved variable [60]. Comparable suppression of labile, redox‐active iron is achievable pharmacologically: intravenous deferoxamine cleared NTBI rapidly in thalassemia major, but the pool reappeared shortly after chelator withdrawal, establishing that continuous exposure is required for sustained control [61]. In transfusion‐dependent myelodysplastic syndromes, 1 year of deferasirox significantly reduced serum ferritin and produced sustained decreases in labile plasma iron, although a discontinuation rate approaching 50% tempers enthusiasm for indefinite chelation in this population [62]. Taken together, these findings position iron sequestration and chelation as an upstream complement to conventional antioxidant supplementation, in that they remove the catalyst of radical generation rather than scavenging the radicals once formed. It should be emphasized, however, that none of these trials measured HSC‐intrinsic redox status or engraftment kinetics as primary endpoints, so the inference that NTBI suppression protects the stem cell compartment remains mechanistically plausible but formally untested.

### 2.6. Current Limitations in Mitigating Oxidative Stress in HSCs

Mitigating oxidative stress in HSC is crucial for their function and therapeutic applications, but several challenges currently limit effective intervention [47]. HSC are rare and predominantly quiescent, which makes their manipulation and genetic modification difficult [63]. Ex vivo culture, often necessary for therapeutic manipulation, can induce oxidative stress, compromising HSC viability, self‐renewal, and long‐term engraftment. This may result in genetic damage, disrupted differentiation processes, and depletion of the stem cell pool [47]. A significant limitation arises from the intrinsic biological properties and challenges associated with ex vivo manipulation [63]. HSC naturally reside in a hypoxic bone marrow niche, and exposure to ambient oxygen during ex vivo procedures increases ROS levels, leading to oxidative stress [64]. This extra‐physiological oxygen shock or stress (EPHOSS) can detrimentally affect HSC function and viability [64]. The quiescent state of HSC also makes them resistant to modification by gene delivery methods that primarily target dividing cells [63]. Furthermore, maintaining HSCs ex vivo without losing their self‐renewal capacity and multipotency remains a significant hurdle, as current culture systems often fail to replicate the native hypoxic and antioxidant‐rich microenvironment [65]. The limited number of HSC available from sources like umbilical cord blood (UCB) also restricts their broader clinical application, as in vitro expansion often results in diminished self‐renewal capacity and reduced regenerative ability [66].

Limitations in gene delivery and editing technologies pose further constraints [67, 68]. While lentiviral vectors are widely used for their ability to transduce nondividing cells, their efficiency can be suboptimal in primitive HSC, often requiring high doses or repeated administrations [67]. This is partly due to intrinsic antiviral restriction factors within the HSC [67]. Nonviral methods, such as electroporation, can introduce high cellular toxicity and cell death, especially with larger plasmid sizes [67]. For gene editing, the CRISPR/Cas9 system offers precision. Still, their efficiency for precise repair via homology‐directed repair (HDR) is low in quiescent HSC compared to nonhomologous end joining (NHEJ), which can lead to gene disruption [67]. Safety concerns related to off‐target effects and potential genotoxicity from double‐strand breaks remain, as these could lead to genomic instability [68]. The lack of reliable preclinical models for assessing long‐term genotoxicity and editing efficiency further complicates clinical development [67]. The complex and multifactorial nature of oxidative stress in HSC adds another layer of difficulty [47]. Both excessively high and inappropriately low ROS levels can impair HSC function, making it challenging to design targeted interventions that maintain the delicate balance [47]. The intricate interactions between HSC and their bone marrow microenvironment, including various cellular components and signaling pathways, further influence oxidative stress regulation, making targeted therapeutic approaches complex [69, 70]. Lastly, technical and economic barriers limit widespread clinical application [47]. The high cost and complexity of ex vivo HSC gene therapy platforms, particularly the cell manufacturing steps, restrict accessibility [47]. Scaling up vector production under good manufacturing practices (GMP) and reducing overall costs are essential for broader clinical translation [47].

## 3. Future Directions for Mitigating Oxidative Stress in HSCs

Future directions in mitigating oxidative stress in HSC involve refining existing technologies and exploring novel strategies to enhance efficacy, safety, and accessibility, with a focus on improved gene delivery and editing, optimized culture conditions, and modulation of endogenous antioxidant pathways. Improved gene delivery and editing technologies are crucial for precise and safe interventions [68]. Efforts are underway to develop safer and more efficient viral vectors, such as self‐inactivating lentiviral vectors (SIN‐LVs), which have a reduced risk of insertional mutagenesis [71]. Foamy virus (FV) vectors are also being explored due to their efficient delivery into HSC and potentially safer integration profiles [63]. Pharmacological agents, such as the cyclic resveratrol trimer caraphenol A, have shown promise in enhancing lentiviral gene delivery efficiency to HSC by modulating antiviral restriction factors, including IFITM2 and IFITM3, thereby augmenting viral core endosomal escape [67]. Advances in gene editing technologies, such as the CRISPR/Cas9 system, are focusing on reducing off‐target effects and improving the efficiency of precise gene modification through HDR [68]. Strategies include transiently increasing the proportion of cells in HDR‐preferred cell cycle phases (S/G2) to enhance precise gene correction [47]. Ribonucleoprotein (RNP) delivery strategies, which directly deliver the Cas9 protein and guide RNA, are being developed to reduce off‐target effects and toxicity by avoiding the introduction of exogenous DNA sequences [47]. Base editing, which enables single‐nucleotide changes without inducing double‐strand breaks, is another promising approach to minimizing genotoxic stress [47].

Optimization of ex vivo culture conditions and niche modulation aims to preserve HSC viability and enhance engraftment [72]. Maintaining HSC under hypoxic conditions (e.g., 3% O_2_) during collection and culture is critical, as it mimics their natural environment and significantly limits ROS production and HSC differentiation, leading to a five‐fold greater recovery of HSC [47]. Administering antioxidants such as NAC or vitamin C in culture media can prevent oxidative damage and enhance the efficiency of cellular reprogramming [47]. Small molecules like cyclosporine A (CsA), which inhibits CypD and the MPTP, have been shown to reduce ROS and enhance the yield and transplantation efficacy of HSC [47]. Biomimetic nanofiber scaffolds are also being developed to replicate the extracellular matrix and provide an ideal physical microenvironment for ex vivo expansion, ensuring HSC self‐renewal and stemness [73].

Targeting endogenous antioxidant pathways directly through gene therapy or pharmacological agents is an emerging strategy [47, 74]. Overexpression of specific antioxidant‐regulating genes or modulation of redox‐sensitive transcription factors within HSC could provide a robust defense against oxidative damage [47]. The Nrf2 pathway is a master regulator of antioxidant enzymes, and its activation can improve redox homeostasis in HSC and niche cells [64]. For instance, the small molecule antioxidant chrysin has been identified to promote HSC self‐renewal by inhibiting ROS‐activated apoptosis and modulating CDK inhibitors [74]. Sirtuins, such as SIRT1 and SIRT3, which regulate mitochondrial metabolic homeostasis and reduce oxidative stress, are also being explored [47]. Upregulating SIRT3, for example, has been shown to improve the regenerative capacity of aged HSC [47]. The FOXO family of transcription factors (FOXO1, FOXO3a, and FOXO4) are crucial for protecting HSC from oxidative damage and maintaining quiescence, and their activity can be modulated to enhance antioxidant defenses [47]. Pharmacological inhibition of pathways, such as p38 MAPK, which is activated by increased ROS and limits HSC lifespan, by agents like SB203580, has been shown to rescue ROS‐induced defects in HSC repopulating capacity [47]. Furthermore, compounds like resveratrol have been shown to inhibit total body irradiation‐induced chronic oxidative stress and senescence in HSC [47].

Understanding and modulating the bone marrow niche is critical, as HSC reside in hypoxic niches where endothelial cells and other neighboring cells help maintain a low ROS state [47]. Future approaches will focus on modulating these interactions to support gene‐modified HSC better and improve their survival and engraftment in vivo [47, 69]. This includes promoting the glycolytic metabolism of endothelial cells, which helps scavenge ROS in their surroundings and contributes to maintaining an ideal site for HSC maintenance [47]. The regulation of vascular permeability within the niche also influences ROS levels, with less permeable arterial vessels associated with lower ROS in HSC [47]. Modulating factors, such as TGF‐β secreted by megakaryocytes and nonmyelinating Schwann cells, which regulate HSC dormancy, also hold potential [47]. Finally, streamlining clinical translation and addressing regulatory and ethical considerations are essential for widespread adoption [71]. This involves scaling up vector production under cGMP‐compliant conditions to make gene therapy more economical and accessible, particularly for prevalent conditions such as hemoglobinopathies [71]. Regulatory frameworks are evolving to align with scientific advances, striking a balance between innovation and patient safety [71]. In parallel, prospective evaluation of iron‐directed interventions, namely, apotransferrin infusion during conditioning or chelator administration in transfusion‐dependent recipients, judged against HSC‐specific redox and engraftment endpoints, constitutes a tractable near‐term avenue precisely because these agents act upstream of radical formation [59–62].

## 4. Conclusion

Oxidative stress arising from an imbalance between ROS generation and the body’s antioxidant defenses has a profound effect on HSC function, impairing their self‐renewal, quiescence, and differentiation while promoting premature aging. In their native hypoxic bone marrow niches, HSC maintain low ROS levels through glycolysis, preserving their long‐term repopulating capacity. However, elevated ROS levels lead to DNA damage, genomic instability, senescence, and apoptosis, contributing to hematopoietic malignancies and transplantation complications. Key molecular pathways, including p38 MAPK and mTOR, are activated by ROS and limit HSC lifespan, whereas transcription factors like FOXO, Nrf2, and sirtuins support antioxidant defense and DNA repair. Therapeutic strategies such as antioxidant supplementation, pharmacological inhibition of ROS‐related signaling, hypoxic ex vivo culture conditions, and gene therapies enhancing endogenous antioxidants show great potential in preserving HSC function and improving transplantation outcomes. Ongoing research into these multifaceted approaches remains crucial for advancing treatments for hematopoietic disorders and improving stem cell‐based therapies.

## Author Contributions

**Dharmaraj Senthilkumar:** writing – review and editing, validation, writing – original draft, visualization, investigation, conceptualization. **Sathish Kumar Venkatachalam**: writing – review and editing, writing – original draft, visualization, validation, conceptualization. **Nallusamy Duraisamy, Suresh Kumar, Zaw Myo Hein, Muhammad Danial Che Ramli, Vasudevan Ramachandran, and Anastasiya Spaska**: writing – review and editing, validation. **Zaw Myo Hein and Anastasiya Spaska**: conceptualization, supervision, funding.

## Funding

The article processing charge was funded by Ajman University.

## Disclosure

All authors read and approved of the final manuscript. No figure element was reproduced or adapted from any previously published work.

## Ethics Statement

The authors have nothing to report.

## Consent

The authors have nothing to report.

## Conflicts of Interest

The authors declare no conflicts of interest.

## Data Availability

No new data have been generated.
